# Spherical Binderless 4A/5A Zeolite Assemblies: Synthesis, Characterization, and Adsorbent Applications

**DOI:** 10.3390/molecules29071432

**Published:** 2024-03-22

**Authors:** Tong Li, Shuangwei Wang, Jinqiang Gao, Ruiqiang Wang, Guifeng Gao, Guangming Ren, Shengnan Na, Mei Hong, Shihe Yang

**Affiliations:** 1 Guangdong Provincial Key Laboratory of Nano-Micro Materials Research, School of Advanced Materials, Peking University Shenzhen Graduate School (PKUSZ), Shenzhen 518055, China; liilitong@163.com (T.L.); jinqiang_gao@pku.edu.cn (J.G.); 2 Ambulanc (Shenzhen) Tech. Co., Ltd., Shenzhen 518108, China; tom.wang@amoulmed.com (S.W.); wayne.wang@amoulmed.com (R.W.); gaoguifeng@amoulmed.com (G.G.); 13798585635@163.com (G.R.); 3 College of Chemistry and Chemical Engineering, Qiqihar University, Qiqihar 161006, China; n15846124725@163.com

**Keywords:** zeolite, microsphere, aging, impregnation, air separation

## Abstract

Zeolite microspheres have been successfully applied in commercial-scale separators such as oxygen concentrators. However, further enhancement of their applications is hampered by the post-synthetic shaping process that formulates the zeolite powder into packing-sized spherical bodies with various binders leading to active site blockage and suboptimal performance. Herein, binderless zeolite microspheres with a tunable broad size range from 2 µm to 500 µm have been developed with high crystallinity, sphericity over 92%, monodispersity with a coefficient of variation (CV) less than 5%, and hierarchical pore architecture. Combining precursor impregnation and steam-assisted crystallization (SAC), mesoporous silica microspheres with a wide size range could be successfully transformed into zeolite. For preserved size and spherical morphology, a judicious selection of the synthesis conditions is crucial to ensure a pure phase, high crystallinity, and hierarchical architecture. For the sub-2-µm zeolite microsphere, low-temperature prolonged aging was important so as to suppress external zeolization that led to a large, single macroporous crystal. For the large 500 µm sphere, ultrasound pretreatment and vacuum impregnation were crucial and facilitated spatially uniform gel matrix dispersion and homogenous crystallization. The obtained zeolite 5A microspheres exhibited excellent air separation performance, while the 4A microspheres displayed ammonium removal capabilities. This work provides a general strategy to overcome the existing limitations in fabricating binder-free technical bodies of zeolites for various applications.

## 1. Introduction

Zeolites, possessing crystalline SiO_4_ and AlO_4_ tetrahedra with well-defined micropores, are important adsorbent and catalytic materials that have greatly expedited the progress of many chemical processes [1]. Zeolites can also be prepared from pure aluminosilicate reactants, as well as from natural resources such as kaolin [2], or wastes such as sludge [3,4], coal fly ash [5], and rice straw [6]. In real industry, technical bodies in shaped form instead of pure nano- or micron-sized powder are used to meet their application ends, mostly with conventional binders into granulates or extrudates [7]. These shaped technical bodies are often multicomponent, consisting of active phases, supports, and various additives [8] with stringent size and morphology requirements. The size requirements of the technical packings are different with various applications. For example, in chromatographic column-based applications, monodisperse micrometer or submicrometer-sized porous microspheres are essential [9]; meanwhile, for a commercial-scale petrochemical process, millimeter or submillimeter uniform bodies are needed [10]. The use of additives such as binders, although achieving their primary function of shaping a mechanically robust body, unavoidably dilutes the key zeolite active sites and blocks accessible pores [11]. Moreover, recent systematic research into the impact of body formations on shaped catalyst properties reveals that uncontrolled processes take place during multiscale shaping such as dealumination, structure rearrangement, and abating [8].

To solve these problems, binderless zeolites have been proposed. Lawson et al. employed a 3D printing technique to fabricate binderless monoliths of zeolite 13X, 5A, and ZSM-5, which exhibited enhanced adsorption properties and comparable mass transport to commercial benchmarks [12]. However, sacrificial biopolymers are still needed to formulate the printing ink that needs to be removed through subsequent high-temperature calcination. The high cost and low throughput of 3D printing also limit its application. Similarly, Liu et al. prepared binderless hierarchical Al-β zeolite in shaped form which exhibited better catalytic performance in the dehydration of 2-(4-ethylbenzoyl)-benzoic acid than the powdered H-form β nanoparticles [13]. The preparation procedure employed traditional extrusion using tetraethyl orthosilicate as a binder, followed by post-treatment with alkaline hydrothermal conversion. Fawaz et al. prepared binderless ZK-4 zeolite microspheres that showed promise in the molecular decontamination of volatile organic compounds [14]. The synthesis utilized macroporous, strongly basic styrene–divinylbenzene anion-exchange resin beads (Dowex MSA-1) as a hard template, which were subsequently calcined to remove the organics. Li et al. prepared monolithic binderless ZSM-5 zeolites using an alkali post-treatment that exhibited improved activity and selectivity in the catalytic cracking of n-hexane [15]. The post-treatment method consisted of strongly blending calcined defective zeolites with silica sol binder, alkaline hydrothermal conversion, and subsequent calcination, which is complex and energy-consuming. Boer et al. synthesized binderless zeolite LTA adsorbents with a macroscopic bead format and hierarchical porosity, reaching higher CO_2_/CH_4_ selectivity and similar CO_2_ adsorption compared to commercial zeolite LTA pellets containing a binder [16]. The synthesis utilized Amberlite IRA-900 anion-exchange resin beads as a hard template, which was subsequently removed using high-temperature calcination. These investigations demonstrated the feasibility of obtaining binderless-shaped technical bodies. Nevertheless, their preparation procedures still involved the use of binders, which were removed during subsequent calcination or post-hydrothermal treatment, leading to high cost and complexity.

To address this issue, we report, herein, a facile one-pot conversion method to directly yield zeolite spheres in technical form, with a wide range from 2 μm to half a millimeter. Spherical particles allow a smooth flow and are preferred for moving beds and packed columns [10]. In a previous study [17], we developed a shape-preserving steam-assisted crystallization (SAC) technique that transformed aluminum and sodium source-impregnated mesoporous silica microspheres into monodisperse zeolite LTA microspheres. The discovered control point for the shape preservation of mother spheres is balancing silica dissolution and zeolite nucleation. However, the size range of the obtained spheres is narrow, namely, only between 10 and 30 μm, and the synthesis of those with smaller diameters failed due to the transition from confined zeolization to external crystallization losing shape control capability. Moukahhal et al. prepared 20 μm hierarchical silicalite-1 zeolite spheres through the pseudomorphic transformation of spherical silica beads with surfactants, also within this size range [18]. In this study, we broke the limit of the previous sphere-preserving size range and revealed more fundamental governing points that promote confined zeolization on a wide scale, scanning from 2 μm to submillimeter, thus opening doors to the direct preparation of technical binderless zeolite spheres without templates or post-treatments.

## 2. Results and Discussion

### 2.1. Synthesis of Downsized Sub-2-μm 4A Microspheres

The use of small sub-2 μm porous silica materials, compared to conventional column packing materials that range from 3 to 5 μm in size, is a promising approach to enhance chromatographic analysis speed, as the van Deemter’s equation shows that the separation efficiency is inversely proportional to the column packing size [19]. Although porous silica microspheres for fast chromatographic separation are rapidly developing with several kinds successfully prepared [20], converting them into corresponding zeolite microspheres has been, so far, unsuccessful. Our previous study [17], unfortunately, found a threshold silica microsphere diameter that limits the shape-reserving transformation into microspherical assemblies of NaA zeolite at around 3 μm. Under the previously selected crystallization conditions, larger cubic crystals with embedded macropores were obtained from porous silica microspheres smaller than 3 μm because the external gel network crystallization dominates below this threshold size value.

**Figure 1:**
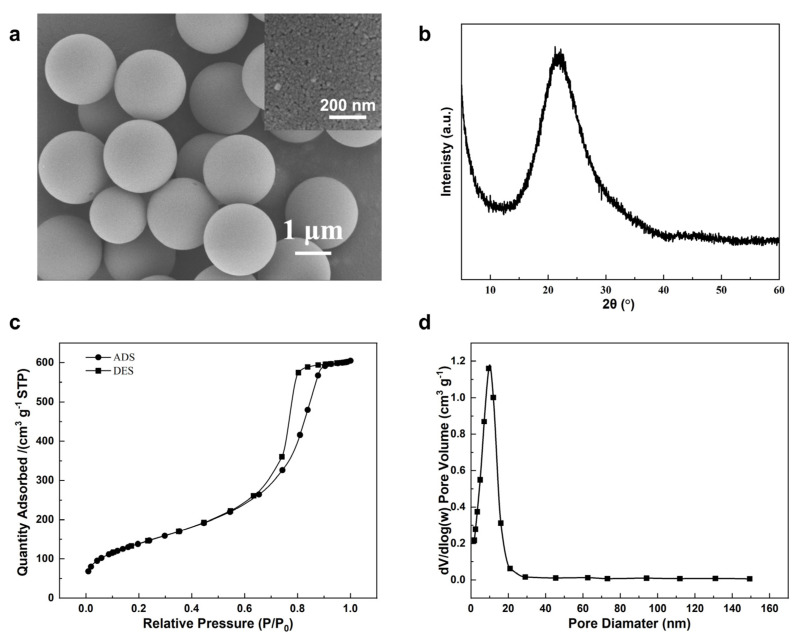
Characterizations of raw material porous S-2 silica microspheres: (**a**) SEM image, inset shows surface features of one single microsphere, (**b**) XRD pattern, (**c**) N_2_ adsorption–desorption isotherms at −196 °C, and (**d**) the corresponding pore size distribution.

**Figure 2:**
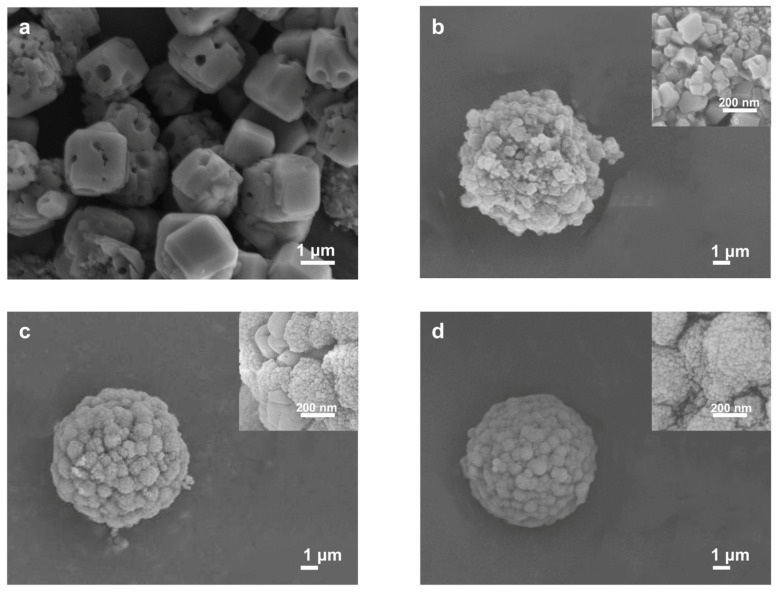
Controlling factors for generating spherical NaA-2: (**a**) Macroporous cubic crystals obtained after aging at room temperature for 10 min; (**b**) Incomplete crystallized spherical NaA assemblies after aging at 0 °C for 10 min; (**c**) Crystallized NaA microspheres after aging at 0 °C for 30 min; (**d**) Monodisperse NaA-2 microspheres after aging at 0 °C for 30 min and with TMAOH. Insets show the surface features of the obtained products.

**Figure 3:**
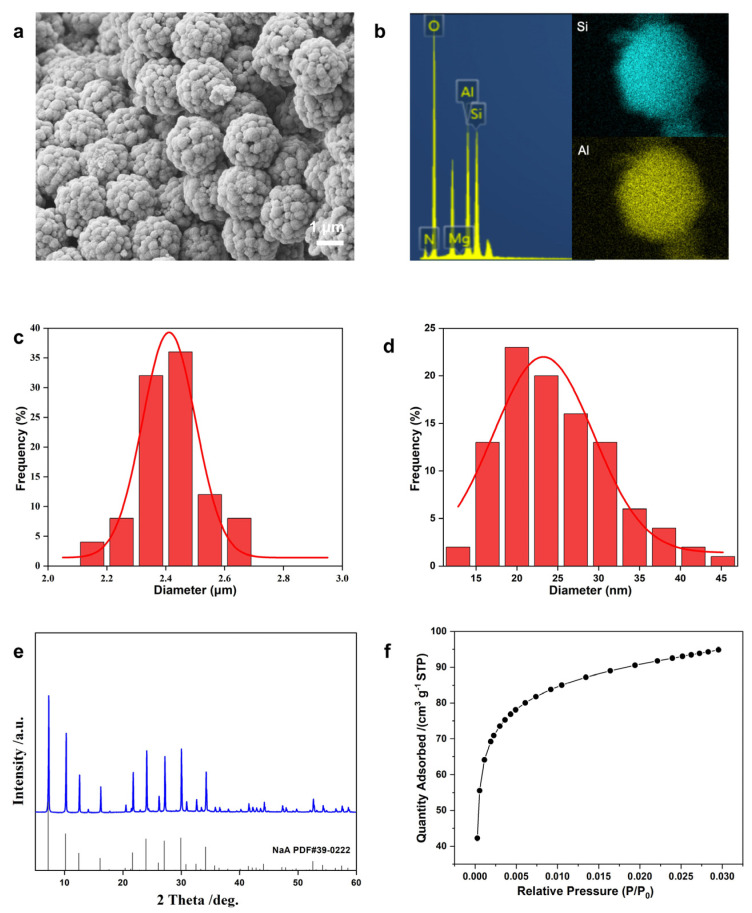
Characterizations of NaA-2 zeolite microspheres: (**a**) SEM image, (**b**) EDS mapping, (**c**) particle size distribution, (**d**) crystal size distribution, (**e**) XRD patterns, (**f**) CO_2_ adsorption isotherms at 0 °C.

To break the size limit, downsizing the zeolite microspheres into the 2 μm region needs careful adjustment of the synthesis conditions, which offer the benefit of enhanced surface sites. We started the synthesis of chromatographic zeolite microspheres using 2 μm silica microspheres S-2, as shown in Figure 1. These silica microspheres are monodispersed with the coefficient of variation (CV) of particle sizes less than 4% (Figure S1). They are highly porous with a BET surface area of 495 m^2^/g and an average mesopore size of 8 nm. Attempts to synthesize zeolite microspheres using our previous method [18] failed, and only nonuniform cubic macroporous crystals larger than 2 μm were obtained instead (Figure 2a), although with high crystallinity and a pure LTA phase (Figure S2a). This indicates that, under previous conditions, external surface nucleation and significant silica dissolution took place, resulting in macropore formation [21]. The suppression of extensive outward silica dissolution relies on the engineering of zeolite nanosized crystals. Mintova and co-workers ingeniously achieved uniformity in the gel precursor by lowering the aging temperature, which afforded rapid transformation into highly crystallized nanozeolites [22]. Adapting this strategy using purposely controlled 0 °C aging temperature, we were able to switch external nucleation to internal transformation, leading to a remarkably lowered single crystalline size to ~60 nm, assembled into semi-spherical shapes (Figure 2b) with maintained high LTA crystallinity (Figure S2b). However, a small amount of undissolved silica still remained, probably due to slowed dissolution at lower temperatures. Further optimization with prolonged aging time led to complete silica transformation and the further reduction of the crystallite size (Figure 2c) while still promoting high crystallinity and pure LTA phase (Figure S2c). However, the crystallite size was not uniform, and particle agglomeration took place, hampering the sphericity and uniformity of the obtained microspheres. To address this issue, organic tetramethylammonium hydroxide (TMAOH), a typical organic template for LTA zeolite synthesis, was added to the precursor that could speed up sol particle condensation [23]. As shown in Figure 2d, monodisperse shape-preserved zeolite microspheres, named NaA-2, with a sphericity of 94% were obtained. As the NaA-2 samples (Figure 2d) possess the desirable phase and morphology, they were further characterized in detail as shown in Figure 3.

Assisted by low-temperature aging, prolonged aging time, and organic TMA^+^ template, the obtained NaA-2 zeolite microspheres possess a preserved particle size of 2.4 μm (Figure 3a) with uniform Si and Al distribution (Figure 3b). The CV of the particle size is as low as 5% (Figure 3c). The LTA crystallites were further reduced to a uniform average size of 25 nm (Figure 3d). These zeolite microspheres are highly crystalline as verified by the XRD patterns (Figure 3e). The Rietveld refinement fitting confirmed the high crystallinity corresponding to the pure zeolite LTA phase (Figure S3).

The nitrogen adsorption isotherm (Figure S4) shows that the micropores of the NaA-2 zeolite microspheres are not accessible to N_2_, which is consistent with previous reports [24], due to the kinetic restriction of the inherent micropore apertures of 0.42 nm from the three-dimensional, small-pore LTA topology that is too narrow for N_2_ molecules to diffuse rapidly in a reasonable equilibrium time at 77 K [25]. Alternatively, carbon dioxide adsorption was obtained at 0 °C (Figure 3f) and revealed a CO_2_ adsorption capacity of 95 cm^3^/g STP, similar to previously reported values [26] for the high-quality LTA-type zeolite.

### 2.2. Synthesis of Enlarged Sub-Millimeter 4A Spheres

**Figure 4:**
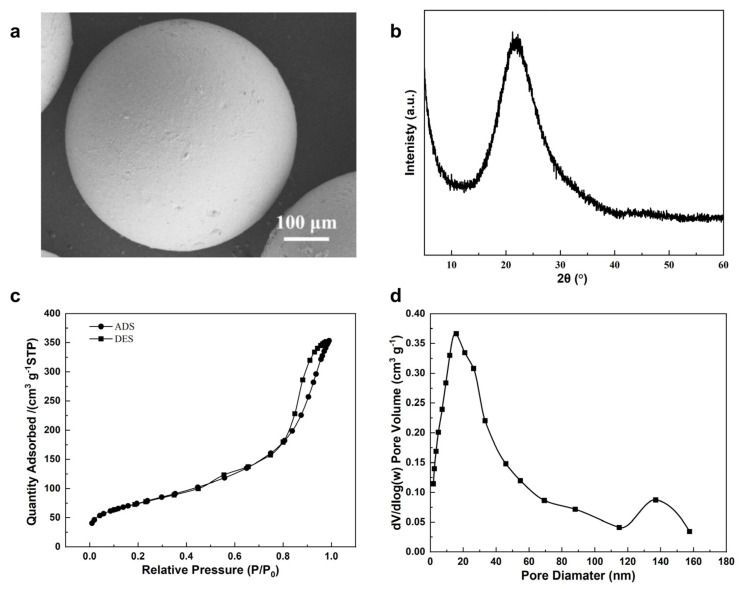
Characterizations of raw material S-500 silica microspheres: (**a**) SEM image, (**b**) XRD pattern, (**c**) N_2_ adsorption–desorption isotherms, and (**d**) the corresponding pore size distribution.

For large-scale industrial adsorption applications, shaped zeolite bodies in the millimeter size range are the preferred form [27]. For smaller medical or personal-scale applications, such as oxygen concentrators, shaped zeolites of submillimeter size are needed [28]. To fulfill the column packing requirement, we chose 0.5 mm silica spheres as the starting material, as shown in Figure 4. These silica microspheres are monodispersed with a coefficient of variation (CV) of a particle size of less than 3% (Figure S5). They are also highly porous with a BET surface area of 263 m^2^/g and an average mesopore size of 8 nm.

**Figure 5:**
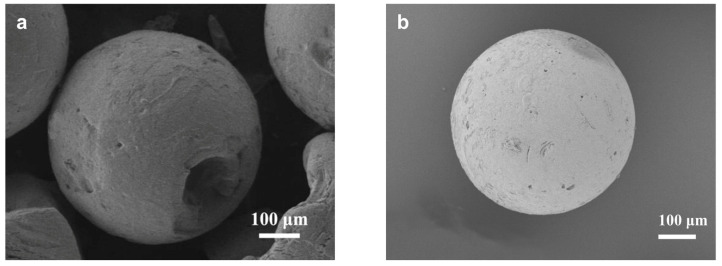
Effect of water removal from impregnated silica on the zeolite external morphology after SAC: (**a**) Dented spheres obtained from impregnated silica directly dried without filtration at room temperature overnight; (**b**) Spherical NaA assemblies from impregnated silica that are filtered and dried at room temperature overnight.

Although our previous SAC method [17] could afford zeolite spheres with a size-preserved 0.5 mm diameter, their surface contained dents (Figure 5a). This suggests dominant local silica dissolution at the dent sites, unmatched by gel network formation. Alkaline solution might react with reactive silica generating viscous sodium silicate which hinders solvent diffusion [29]. To avoid early silica dissolution, the impregnated silica spheres were filtered before drying at room temperature overnight. This intermediate filtration process mitigates the liquid water phase, slowing down the mass transportation of silicate species and promoting silica-alumina gel homogeneity during the reorganization process. Adopting this method, preserved zeolite microspheres with undented surfaces were obtained (Figure 5b).

**Figure 6:**
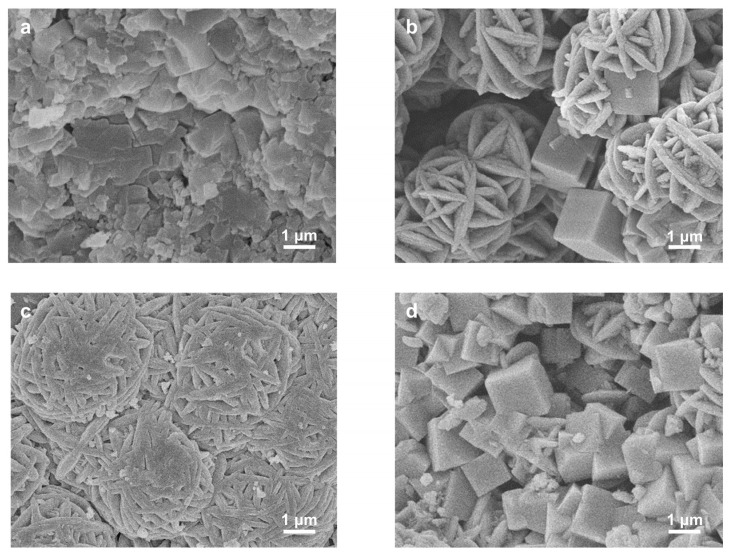
Controlling factors for generating spherical NaA-500 with spatially uniform crystal assembly: (**a**,**b**) Effect of vacuum infiltration during silica impregnation showing non-uniform zeolite microstructure obtained without vacuum infiltration of (**a**) internal and (**b**) external surface; (**c**,**d**) Effect of ultrasound pretreatment during silica impregnation on the zeolite microstructure showing expedited crystallization leading to SOD formation at 8 h.

For the sub-millimeter silica microsphere, zeolite growth uniformity throughout the entire sphere is critical but remains challenging. Conventional impregnation at room temperature, as we used previously, was spatially incomplete, and the internal silicon source became inaccessible to the aluminum and alkali source; thus, it cannot be fully dissolved and crystallized. Arising from the different gel networks, the inner and outer surfaces of the sphere grow unevenly. Figure 6a reveals that the inner part of this microsphere contains undissolved amorphous silica besides the crystallized cubic LTA zeolites, and Figure 6b shows that the external surface contains both cubic LTA and rugged spherical SOD crystals. Thus, the outer surface grew extensively, inducing phase transformation from LTA to thermodynamically more stable SOD crystals due to the Ostwald rule of stages [30], while the inner surface was not completely crystallized, leaving undissolved silica. The EDS mapping results (Figure S6) indicate an uneven Si/Al ratio within the zeolite spheres. The external surface possessed a Si/Al of 1, and the inner core had a much higher value of 1.4. The extensive radical transport resistance obviously brings about resistance to the sodium aluminate and hydroxide penetration, consistent with the hypothesis obtained from sub-nanometer precursors that exterior surfaces are more energetically favorable for nucleation compared to the interior ascribed to the similar confinement effect [31], although at different size scale.

To effectively promote homogeneous nucleation and speed up crystallization, we employed vacuum impregnation to induce forced infiltration and ultrasound pretreatment to take advantage of the acoustic cavitation in sonochemistry [32]. Without ultrasonic pretreatment, the obtained zeolite spheres were, spatially, not uniform, even with the aid of vacuum impregnation (Figure 6c,d). By employing ultrasonication to the suspension of the silica microsphere in impregnation sodium aluminate, we could disperse more evenly the aluminosilicate precursor. This helps the synthesis of more uniform highly crystalline LTA zeolite microspheres. Another benefit of ultrasound pretreatment is the greatly reduced crystallization time [33]. Ultrasound pretreatment accelerated zeolite nucleation and growth so that the required SAC time for crystallization into highly crystalline LTA zeolite microspheres is only 6 h. Beyond this time, SOD polymorph gradually formed, and complete solid phase conversion to SOD microspheres took place at an SAC time of 8 h (Figure S7). Without ultrasound pretreatment, the crystallization time to obtain LTA zeolite microspheres was 10 h.

**Figure 7:**
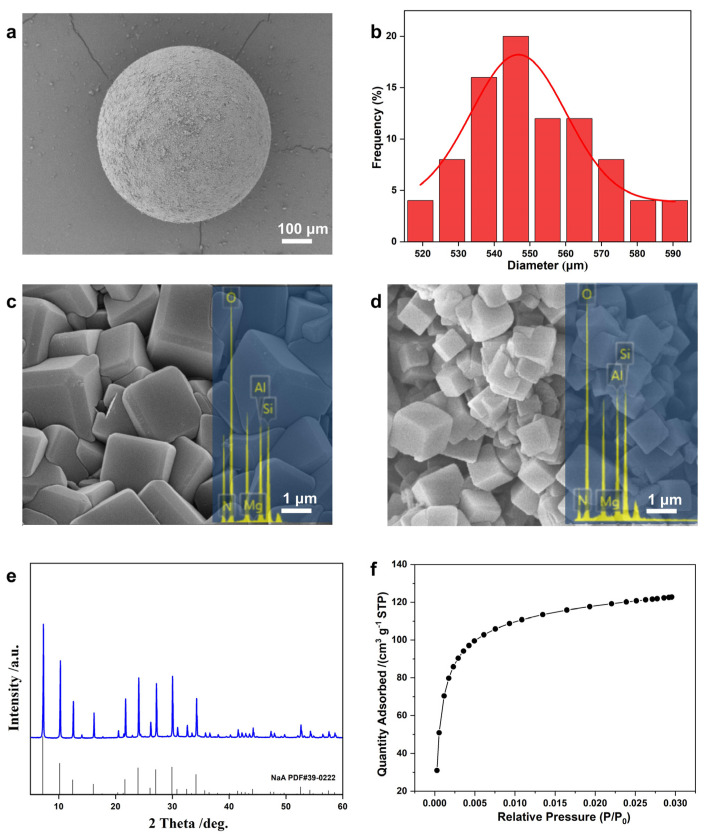
Characterizations of NaA-500: (**a**) SEM overview of a single sphere, (**b**) particle size distribution with a CV of 4%, (**c**,**d**) SEM images and EDS mappings of (**c**) external surface and (**d**) interior, (**e**) XRD pattern, (**f**) CO_2_ adsorption isotherms at 0 °C.

Therefore, with the aid of ultrasound pretreatment to promote rapid synthesis, vacuum impregnation to ensure precursor spatial uniformity throughout the entire silica spheres, and prefiltration before SAC to control the water content of solid precursor, highly crystalline pure-phase LTA microspheres, named NaA-500, were obtained after the proper adjustment of Al content, as shown in Figure 7. The NaA-500 spheres were monodispersed with a diameter of 500 µm, CV of 4%, and sphericity of 92% (Figure 7a,b). These LTA zeolites were uniform throughout the crystal exterior (Figure 7c) and inside (Figure 7d) regarding crystal morphology and elemental composition. XRD pattern (Figure 7e) and Rietveld refinement fitting (Figure S8) confirmed the formation of pure-phase LTA crystals with slight crystal orientation. Due to the kinetic restrictions at cryogenic temperature, nitrogen adsorption is of limited value for the characterization of NaA zeolites [34]. Mercury intrusion porosimetry analysis (Figure S9a) demonstrated mesopores around 30 nm (Figure S9b), likely arising from intracrystalline space within the microsphere, leading to facilitated transport. Carbon dioxide adsorption at 273 K (Figure 7f) was utilized to access the relatively small micropore of NaA-500, revealing the CO_2_ adsorption capacity of 123 cm^3^/g STP, with calculated micropore volumes of 0.23 cm^3^/g, similar to the NaA-2 zeolite microspheres.

### 2.3. Air Separation and Waste Removal Performance

The morphological features of NaA-2 and NaA-500 both show the spherical assembly of cubic LTA crystallites, thus showing a hierarchical structure. Table 1 summarizes the structural and textural properties of the parent silica and the transformed zeolite microspheres according to previously published methods [25,35]. The high degree of intracrystalline mesoporosity is beneficial for packing-related applications. For some adsorption applications, the cation exchange of zeolite NaA (4A zeolite) is important for constructing 5A adsorbent with a micropore size of ~0.5 nm [36]. This is also true for creating air separation adsorbent [37]. The Si/Al ratio of almost 1 and the stronger polarization effect of Ca^2+^ compared to that of Na^+^ offer an additional attractive interaction [38].

**Figure 8:**
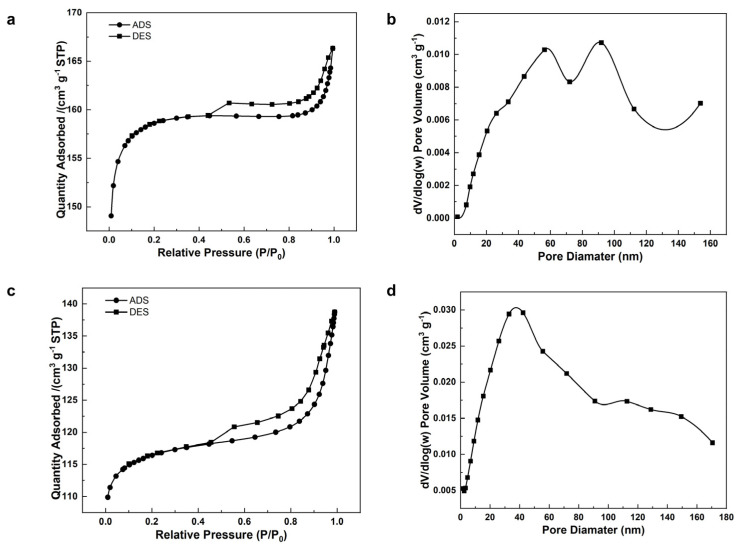
Characterization of CaA-2 and CaA-500: (**a**) N_2_ adsorption–desorption isotherms at −196 °C and (**b**) the corresponding pore size distribution of CaA-2; (**c**) N_2_ adsorption–desorption isotherms at −196 °C and (**d**) the corresponding pore size distribution of CaA-500.

Both NaA-2 and NaA-500 were exchanged with calcium cation to prepare CaA-2 and CaA-500 zeolite spheres with different diameters. The Ca^2+^ could open the micropore aperture, making them accessible to nitrogen gas, and the N_2_ adsorption–desorption isotherms are listed in Figure 8. Due to the inherent micropores and intracrystalline mesopores, both CaA-2 and CaA-500 are also highly porous with BET surface areas of 501 m^2^/g and 357 m^2^/g, with an average mesopore size of 32 nm and 24 nm, respectively. Compared to the hierarchical Ca-form LTA zeolites obtained by using amino acids with S_BET_ between 445 and 492 m^2^/g and a mesopore size between 18 and 19 nm [39], the BET surface areas are similar while the mesopore sizes are larger, because the secondary pores in the CaA-2 and CaA-500 spherical assemblies are intercrystalline, unlike the intracrystalline mesopores generated using organic mesoporogens.

**Figure 9:**
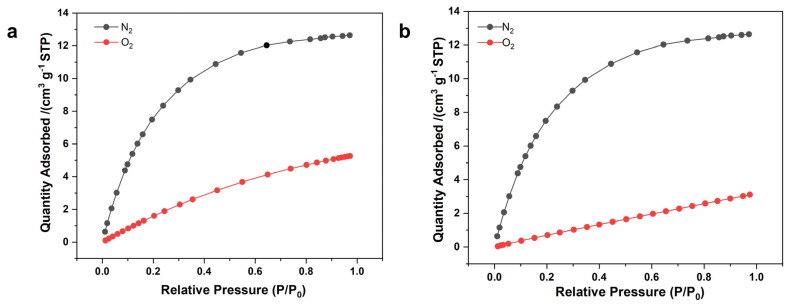
N_2_ and O_2_ adsorption isotherms at 0 °C of (**a**) CaA-2 and (**b**) CaA-500.

They were tested for nitrogen and oxygen adsorption at 0 °C (Figure 9). Both CaA-2 and CaA-500 microspheres exhibited an almost identically high nitrogen adsorption capacity of ~13 cm^3^/g at 0 °C and 99 kPa, similar to the previously reported CaA-30-10 microspheres with a diameter of 30 μm, measured using a volumetric method under identical conditions, and ~10% higher than that reported in the literature [40]. The uptake of O_2_ at *P*/*P*_0_~0.98 was 3~5 cm^3^/g at 0 °C. The enlargement and downsizing of the zeolite microspheres did not affect the nitrogen adsorption capacity and N_2_/O_2_ separation factor, likely benefiting from the hierarchical microstructure and high crystallinity of the binder-free assemblies, which hold great potential for high-performance oxygen concentrators.

**Figure 10:**
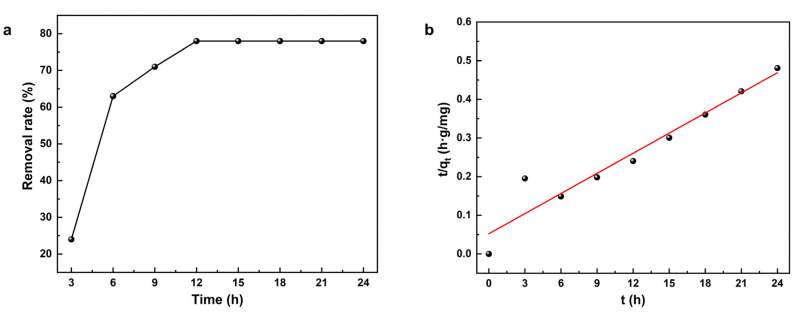
Ammonia nitrogen adsorption kinetics using NaA-500 zeolite microspheres: (**a**) removal profile; (**b**) pseudo-second-order kinetics plot.

To further explore the potential of NaA-500 zeolite microspheres as adsorbent, we conducted the removal of ammonia nitrogen, which is a risk factor to aquatic ecosystems due to its toxicity for various aquatic organisms [41]. At room temperature, for treating 500 mg/L ammonia nitrogen with a pH of 7, the adsorption kinetic curve for ammonia nitrogen adsorption on as-synthesized NaA-500 zeolite microsphere is shown in Figure 10a, revealing fast and efficient removal that achieves a removal rate of over 70% in 9 h. Then, it became saturated and leveled off at close to 80% afterward. To further understand the adsorption kinetics, modeling the experimental data with the pseudo-first-order and pseudo-second-order kinetic equation was conducted. Linear fitting results and the corresponding R-squared (R^2^) statistics demonstrated that the pseudo-second-order kinetic model can describe the adsorption kinetics more accurately (R^2^ = 93%) than the pseudo-first-order kinetic model (R^2^ = 64%). The pseudo-second-order modeled lines in Figure 10b fitted well with the experimentally measured data, indicating that the adsorption of ammonia nitrogen by an NaA-500 zeolite microsphere may not be determined by a single factor, and the chemisorption may be one of the influencing factors [42]. Fitted with the pseudo-second-order model, the calculated equilibrium ammonia adsorption capacity q_e_ was 64.52 mg/g, corresponding to a removal rate of 78% in a single batch, which is similar to modified bentonite [43]. To determine whether intraparticle diffusion is the rate-limiting step, we performed an intraparticle diffusion model analysis, as shown in Figure S10. The model plot of adsorption amount q_t_ vs. t_1/2_ was not a single linear line passing through the origin; thus, in the hierarchical assembly, as in NaA-500, intraparticle diffusion is not the rate-limiting step. The binder-free 4A zeolite microspheres, having a large uniform size of 500 µm, are easy to handle and can be simply separated from water by sedimentation (Figure S11). Combined with low-cost and facile scale-up, the NaA-500 zeolite microspheres hold great potential for the advanced treatment of ammonia nitrogen wastewater.

## 3. Materials and Methods

### 3.1. Chemicals and Reagents

The 2-micron silica microspheres were provided by Suzhou Nanomicro Technology Co., Ltd. (Suzhou, China), and the submillimeter-sized silica spheres were purchased from Qingdao Xinchanglai Silicone Co., Ltd. (Qingdao, China). Sodium hydroxide (NaOH), sodium aluminate (NaAlO_2_), tetramethylammonium hydroxide (25% solution), inorganic salts of calcium chloride (CaCl_2_), and calcium hydroxide (Ca(OH)_2_) were purchased from J&K (Beijing, China). Ammonia as N IC Standard (100 mg/L) and Nessler Reagent were purchased from Aladdin (Shanghai, China). All solvents and salts were obtained at their analytical grade and used without further treatment. Gases of nitrogen, oxygen, and carbon dioxide were obtained from Xiangheng Gas (Tai’an, China), all with purity over 98%. Water was supplied by a Barnstead Nanopure water system (Thermo Scientific, Waltham, MA, USA, 18.3 MΩ cm).

### 3.2. Synthesis of Zeolite Microspheres

#### 3.2.1. Synthesis of 2-Micron-Sized LTA Zeolite Microspheres

Porous silica microspheres with a diameter of 2 µm, supplied by Suzhou Nanomicro Technology Co., Ltd. (denoted as S-2), were used as a sacrificial template for the transformation into crystallized zeolite microspheres using steam-assisted crystallization. In an optimized synthesis procedure, 66 mg NaOH and 136 mg NaAlO_2_ were sonicated in 2 mL deionized water and stirred until clear, followed by the addition of 0.2 mL tetramethylammonium hydroxide (TMAOH) with a volume fraction of 25% and mixing with 100 mg SiO_2_ microspheres under strong stirring at 0 °C for 30 min. The silica microspheres were impregnated in the alkaline aluminate solution for 30 min under vacuum. Then, the mixture was placed in a PTFE holder and dried at room temperature for 9 h until the surface was completely dry. The Teflon holder was then placed in a 100 mL autoclave containing 15 mL of water at the bottom and steam-assisted crystallization was conducted at 110 °C for 10 h to ensure full evaporation of the bottom water to create a desired steam environment. After crystallization, the autoclave was cooled to room temperature with cold water. The resulting powder collected with a centrifuge was washed three times with deionized water until the pH of the supernatant was close to 7, dried at 60 °C for more than 12 h, and calcined at 550 °C for 6 h. These LTA samples were named NaA-2.

#### 3.2.2. Synthesis of Millimeter-Sized LTA Zeolite

Millimeter-sized commercial silica gel was used as a sacrificial template for the transformation into crystallized zeolite microspheres using the SAC method. In an optimized synthesis procedure, 66 mg NaOH and 165 mg NaAlO_2_ were sonicated in 2 mL deionized water and stirred until clear, followed by the addition of 100 mg of 500 μm SiO_2_ microspheres (denoted as S-500) and stirring for 30 min, with intermittent ultrasonication (KQ-250DE, Kun Shan Ultrasonic Instruments Co., Ltd., Shanghai, China) for 5 min. To ensure sufficient impregnation of the silica spheres in the alkaline aluminate solution, vacuum conditions were employed, and then the fully impregnated silica spheres were aged for 9 h, filtered, and dried. The Teflon holder with the fully impregnated silica spheres on top was then placed in a 100 mL autoclave containing 15 mL of water at the bottom and steam-assisted crystallization was conducted at 110 °C for 6 h. After crystallization, the autoclave was cooled to room temperature with cold water. The resulting solid collected with a centrifuge was washed three times with deionized water until the pH of the supernatant was close to 7 and dried at 60 °C for more than 12 h. These LTA samples were named NaA-500.

#### 3.2.3. Calcium Cation Exchange

The calcium cation exchange converts the NaA (4A) zeolites to CaA (5A) zeolites. Calcium cation exchange was conducted by mixing 500 mg of LTA zeolite spheres with a 25 mL solution of 1 M calcium chloride solution, the pH of which was adjusted to 9 with 1 M calcium hydroxide. After stirring slowly for 6 h at 80 °C in a 500 mL sealed glass flask, the Ca^2+^ exchanged zeolite was centrifuged and washed with deionized water. This Ca^2+^ exchange procedure was repeated five times and then the collected sample was dried at 60 °C overnight in a vacuum. These samples were named CaA-2 and CaA-500.

#### 3.2.4. Ammonia Nitrogen Adsorption Experiments

The initial concentration of ammonia nitrogen in the experiment was selected to be 500 mg/L. The adsorption experiment was performed by stirring 1 g zeolite spheres in 100 mL of simulated waste ammonium aqueous solutions at room temperature with a pH of 7 for 24 h; then, the suspension was separated through sedimentation. The concentration of the supernatant was measured using a UV-vis spectrophotometer (Shimadzu, Kyoto, Japan, UV-2600). Nessler’s reagent method was used to determine the ammonia nitrogen concentration. The adsorption capacity *Q_e_* (mg/g) for ammonia nitrogen was calculated with the following equation:

(1) $$Q_{e}=\frac{V(C_{0}-C_{e})}{m}$$

where $C_{0}$ is the initial concentration (mg/L), $C_{e}$ is the equilibrium concentration (mg/L), V is the solution volume (L), and m is the zeolite mass (g).

### 3.3. Characterization

Powder X-ray diffraction (XRD) patterns of LTA zeolites were recorded using a Rigaku Smartlab 3000w (Tokyo, Japan) in the diffraction angle range 2θ = 5–50° with Cu Ka radiation (λ = 1.5418 Å at 40 KV, 40 mA). Scanning electron microscopy (SEM) was performed using a JEOL JSM-7800F electron microscope (Akishima, Japan) operated at 5.0 KV. Based on the SEM images, the particle size distribution and average particle diameter were obtained from the measurement of at least 100 particles, and the sphericity was calculated using Image-Pro Plus 6.0. Si/Al ratios of the synthesized samples were measured using a Falcon energy-dispersive spectrometer (EDS, Oxford Instruments X-Max^N^, 51-XMX1112, Oxford, UK). Nitrogen desorption-adsorption isotherms at −196 °C were obtained using a Micromeritics Tristar II 3020 v1.03 analyzer (Norcross, GA, USA). Nitrogen, oxygen, and carbon dioxide adsorption isotherms for evaluating air separation performances were conducted at 0 °C. For all these gas sorption experiments, the powder samples were degassed at 190 °C in vacuo for at least 6 h before measurements. Based on nitrogen gas adsorption data at −196 °C, the Brunauer–Emmett–Teller (BET) surface area (S_BET_) was calculated in the range of 0.05 < *P/P*_0_ < 0.3. The mesopore size distributions and mesopore diameter (D_meso_) were determined using the Barrett–Joyner–Halenda (BJH) model from the desorption branch calculated using MicroActive for asap 2460 software. Total pore volume (V_total_) was calculated as the amount of N_2_ adsorbed at *P*/*P*_0_ = 0.98. From CO_2_ adsorption at 0 °C, the Dubinin–Radushkevich (DR) equation was used to assess the micropore volume.

## 4. Conclusions

In summary, the transformation of silica microspheres into zeolite microspheres in a wide range between 2 µm and 500 µm was achieved via an optimized SAC method; moreover, they are distinctly different from commercial adsorbents that are formulated with binders and additives. Properly balancing precursor impregnation, silica dissolution, and internal and external zeolization allows the homogenous crystallization of shape- and size-preserved zeolite microspheres within a broad range. The binderless zeolite microspheres exhibited excellent and almost identical N_2_ and O_2_ adsorption capacities with a high nitrogen adsorption capacity of ~13 cm^3^/g and an oxygen uptake of 3~5 cm^3^/g at 0 °C, 99 kPa, regardless of the particle diameter thanks to the hierarchical porous structure. They also showed their capacity for ammonia nitrogen removal. Overall, this work provides a prospective strategy for promoting the development of binderless zeolite technical bodies on an industrial scale.

## Figures and Tables

**Table 1:** Structural and textural properties of parent silica and transformed zeolite microspheres.

	a\AA ^a^	Si/Al ^b^	Si/Al ^c^	V_micro_ ^d^ (cm^3^/g)	V_total_ ^e^ (cm^3^/g)	S_BET_ ^f^ (m^2^/g)
Si-2	-	-	-	-	0.93	495
Si-500	-	-	-	-	0.21	263
NaA-2	25.020	1.01	1.00	0.19	0.97 ^g^	501 ^g^
NaA-500	24.996	1.07	1.00	0.23	0.54 ^g^	357 ^g^

^a^ Lattice parameter obtained from powder XRD pattern using the Breck and Flanigen equation; ^b^ Si/Al ratio calculated from XRD patterns; ^c^ Si/Al ratio obtained from chemical analysis using ICP; ^d^ Micropore volumes obtained from CO_2_ adsorption at 0 °C using the Dubinin−Radushkevich (DR) equation; ^e^ Total pore volume calculated as the amount of N_2_ adsorbed at −196 °C at *P/P_0_* = 0.98; ^f^ BET surface area obtained from N_2_ adsorption isotherm in a relative pressure range of 0.05–0.30; ^g^ the samples were ion-exchanged with calcium.

## Data Availability

Data are available upon request.

## Supplementary Materials

The following supporting information can be downloaded at: https://www.mdpi.com/article/10.3390/molecules29071432/s1, Materials characterization data: Figures S1–S10; Image of waste removal: Figure S11.
