# Supplementary material for: Spherical Binderless 4A/5A Zeolite Assemblies: Synthesis, Characterization, and Adsorbent Applications
Source: Molecules. 2024 Mar 22;29(7):1432. doi: 10.3390/molecules29071432 (PMC11012900; doi:10.3390/molecules29071432)
Supplement: Supplementary file 1 [file molecules-29-01432-s001.zip › molecules-2863683-supplementary.pdf]

Spherical Binderless 4A/5A Zeolite Assemblies: Synthesis, Characterization, and Adsorbent Applications

Tong Li [1,†], Shuangwei Wang [2,†], Jinqiang Gao [1], Ruiqiang Wang [2], Guifeng Gao [2], Guangming Ren [2],
Shengnan Na [3], Mei Hong [1,*] and Shihe Yang [1,*]

[1] Guangdong Provincial Key Laboratory of Nano-Micro Materials Research, School of Advanced Materials, Peking University Shenzhen Graduate School (PKUSZ), Shenzhen 518055, China; liilitong@163.com (T.L.); jinqiang_gao@pku.edu.cn (J.G.)
[2] Ambulanc (Shenzhen) Tech. Co., Ltd., Shenzhen 518108, China; tom.wang@amoulmed.com (S.W.); wayne.wang@amoulmed.com (R.W.); gaoguifeng@amoulmed.com (G.G.); 13798585635@163.com (G.R.)
[3] College of Chemistry and Chemical Engineering, Qiqihar University, Qiqihar 161006, China; n15846124725@163.com
* Correspondence: hongmei@pku.edu.cn (M.H.); chsyang@pku.edu.cn (S.Y.)
† These authors contributed equally to this work.

Supplementary figures

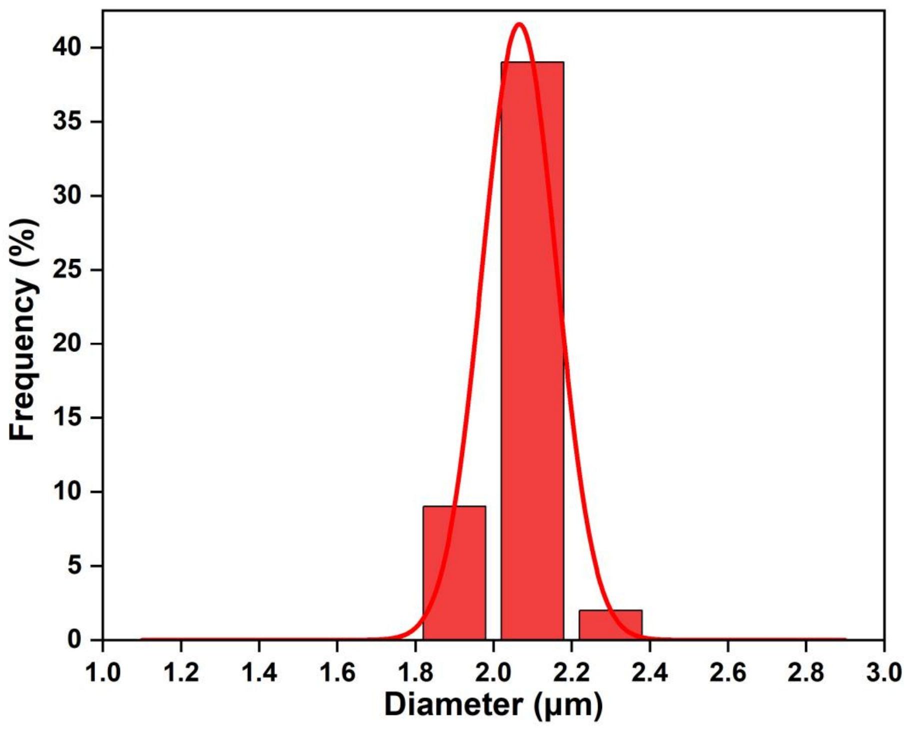

Figure S1. Particle size distribution of S-2 silica microspheres.

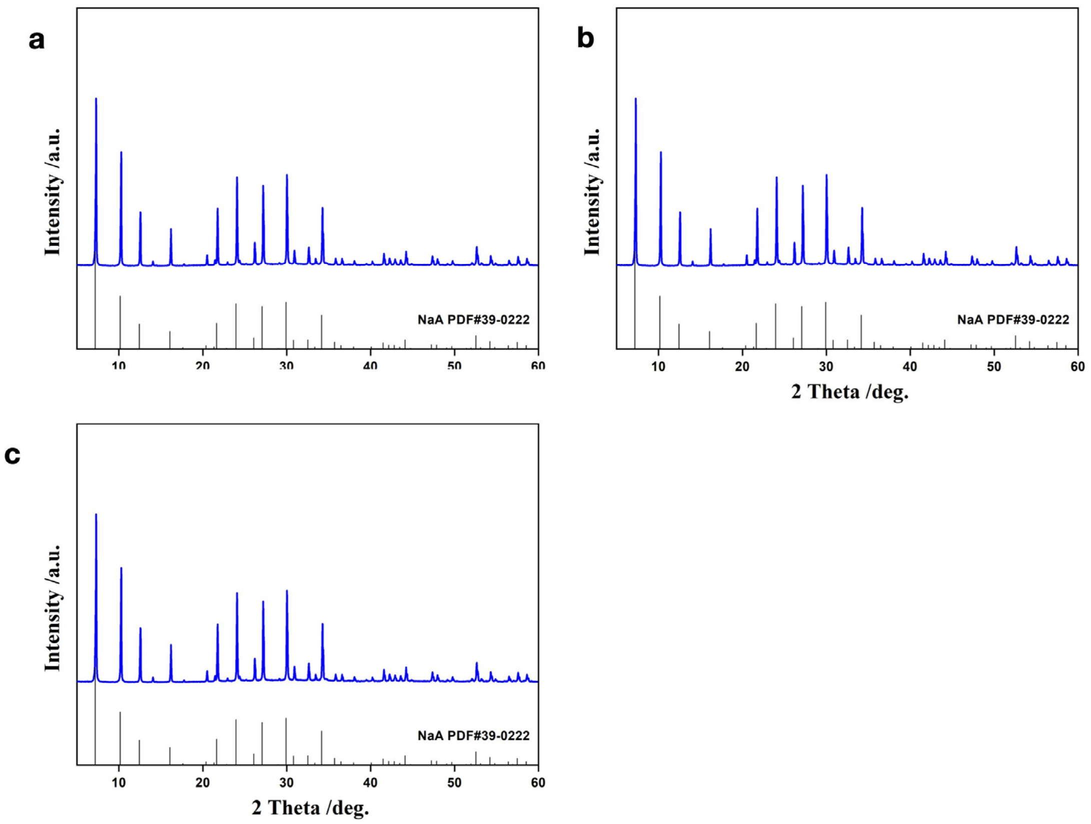

Figure S2. XRD pattern of zeolite microspheres synthesized from S-2 under different pretreatments corresponding to those of Figure 2: (a) Macroporous cubic crystals obtained by aging at room temperature for 10 min; (b) Incompletely crystallized spherical NaA assemblies by aging at 0°C for 10 min; (c) Crystallized NaA microspheres by aging at 0°C for 30 min.

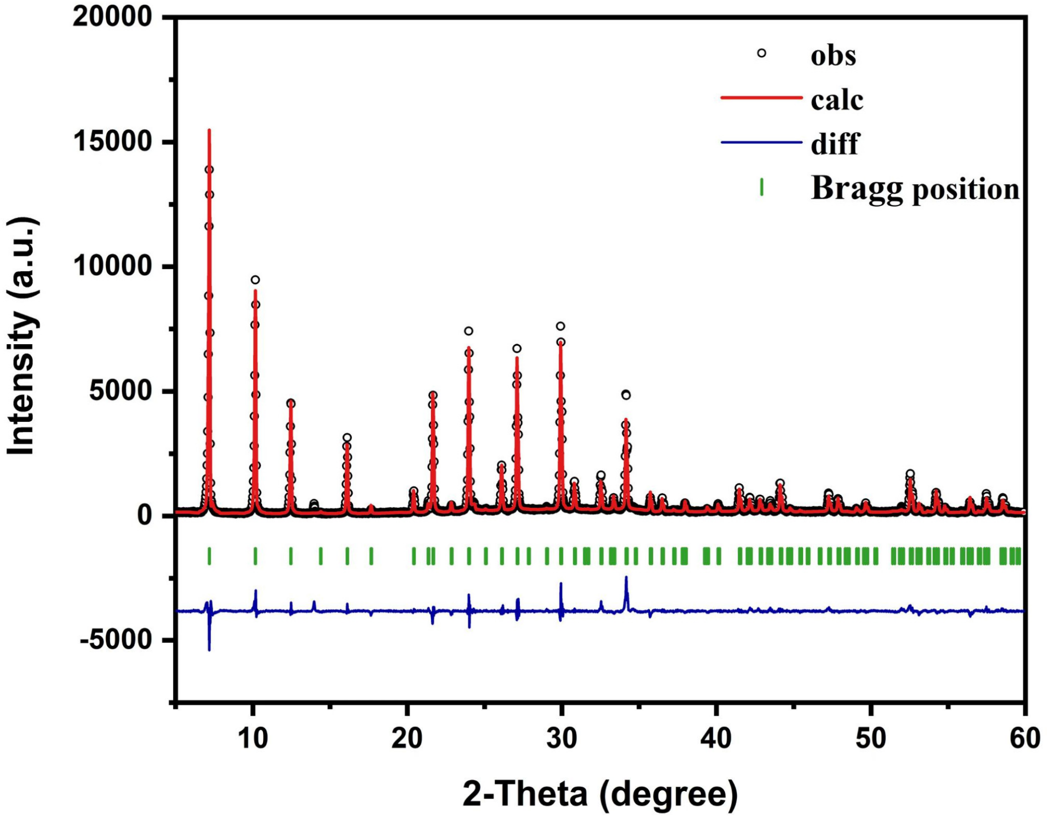

Figure S3. Rietveld refinement fitting on the room temperature powder XRD pattern of NaA-2.

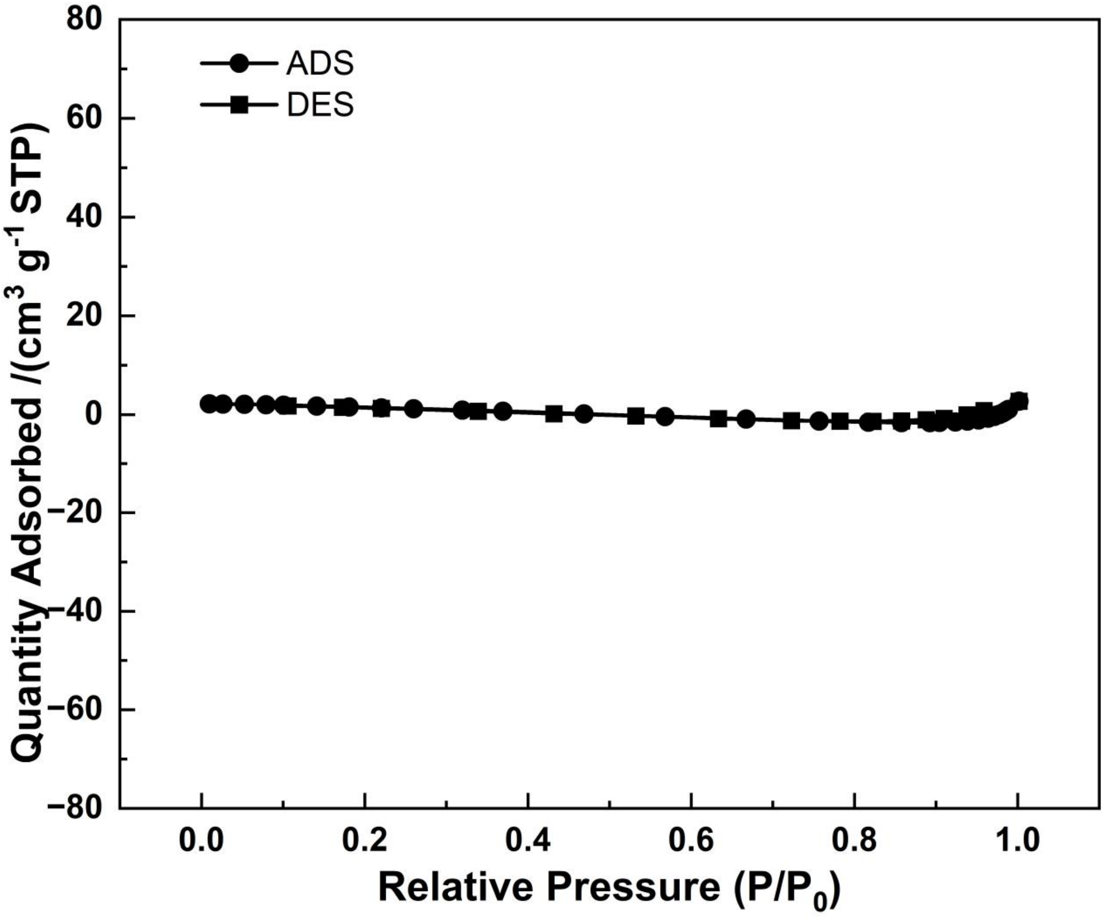

Figure S4. N_2 adsorption-desorption isotherms at -196 ^{o}C of NaA-2 zeolite microspheres.

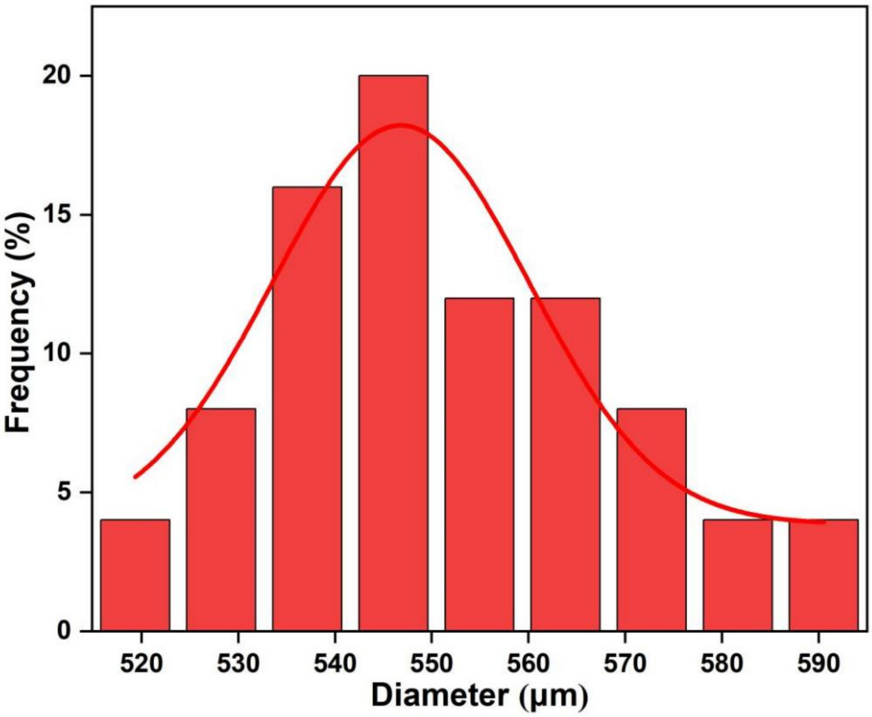

Figure S5. Particle size distribution of S-500 silica microspheres.

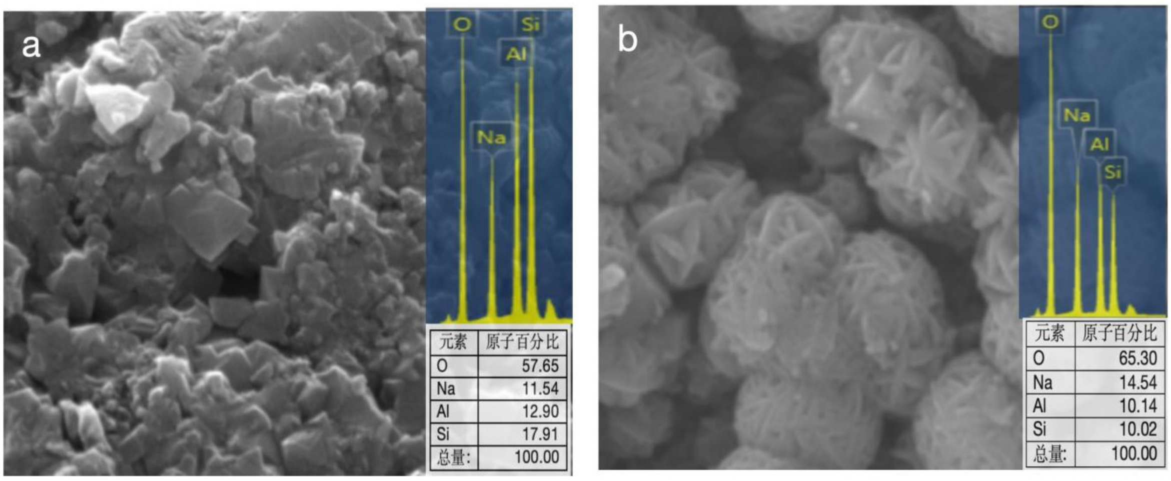

Figure S6. The EDS mapping results of NaA spheres obtained from conventional impregnation indicating uneven Si/Al ratios: (a) inner core, (b) external surface.

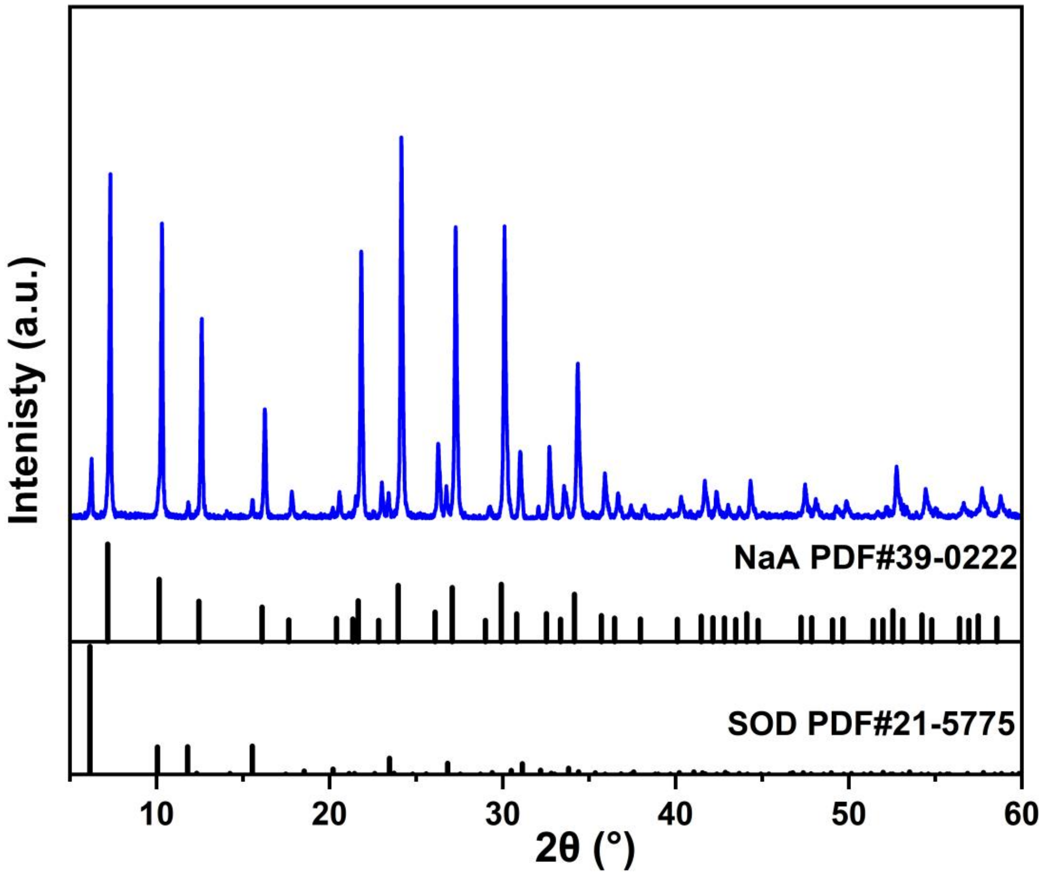

Figure S7. XRD pattern of zeolite microspheres synthesized with ultrasound pretreatment and SAC for 8 h showing SOD impurity.

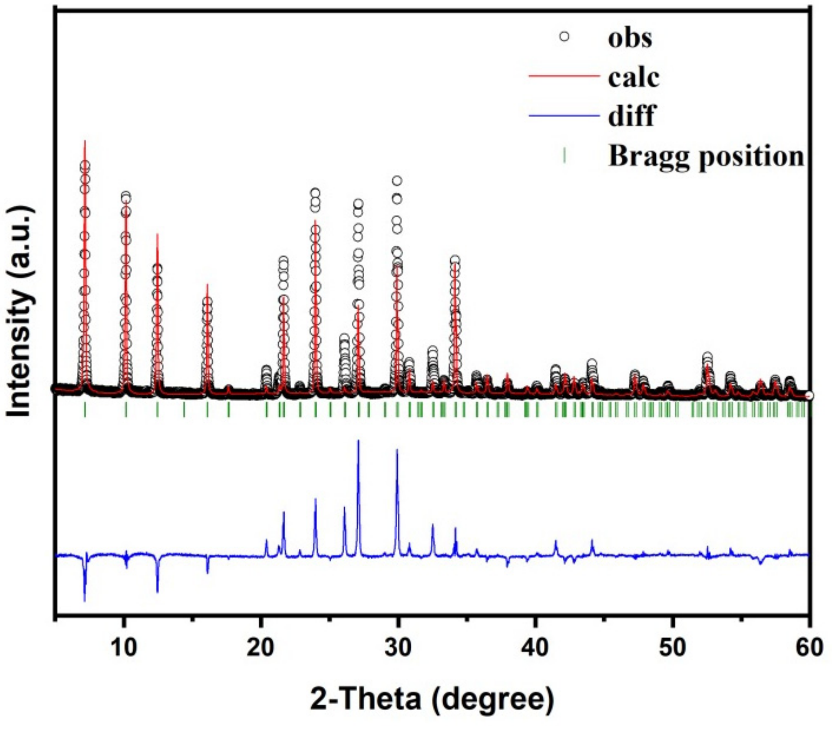

Figure S8. Rietveld refinement fitting on the room temperature powder XRD pattern of NaA-500

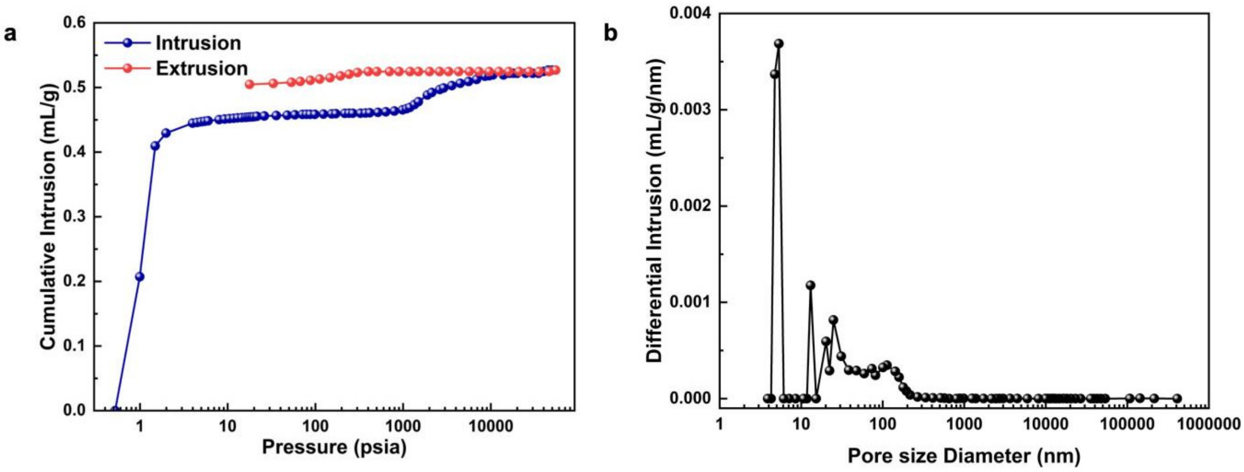

Figure S9. (a) mercury intrusion porosimetry, and the corresponding (b) pore size distribution of NaA-500.

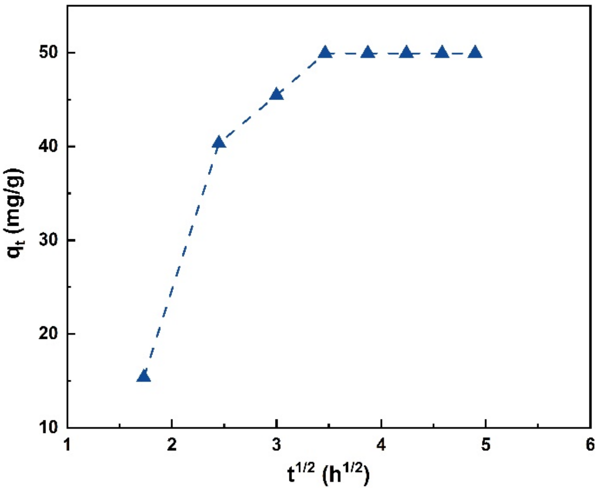

Figure S10. Plot of q_t versus $t^{1/2}$ for ammonia nitrogen adsorption on NaA-500 zeolite microsphere for analyzing intraparticle diffusion.

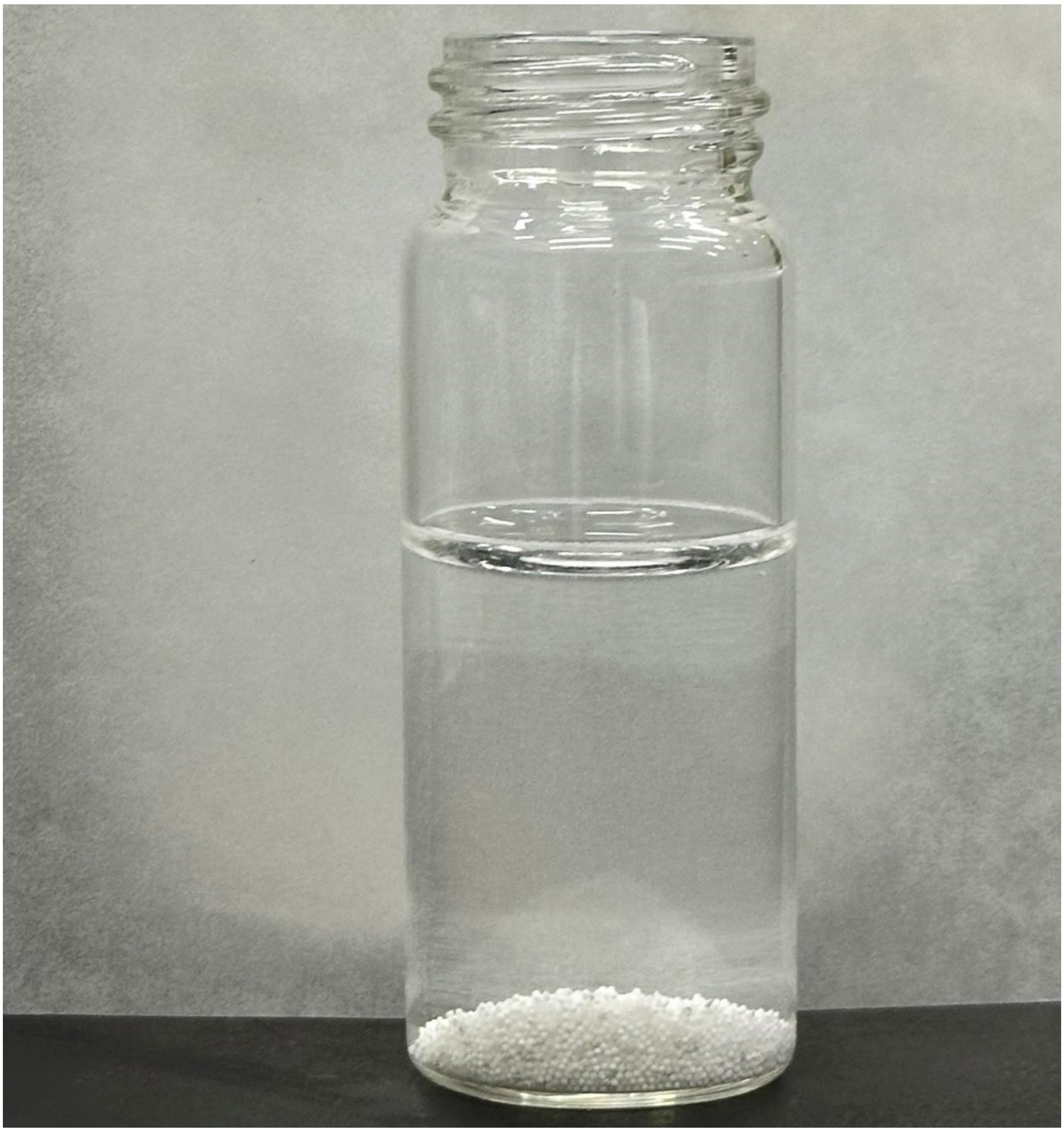

Figure S11. Digital photo of the NaA-500 sample for removal of ammonia nitrogen that could be separated by sedimentation.
